# Data Challenges for Externally Controlled Trials: Viewpoint

**DOI:** 10.2196/43484

**Published:** 2023-04-05

**Authors:** Russanthy Ruthiran Velummailum, Chelsea McKibbon, Darren R Brenner, Elizabeth Ann Stringer, Leeland Ekstrom, Louis Dron

**Affiliations:** 1 Cytel, Inc Vancouver, BC Canada; 2 Department of Oncology University of Calgary Calgary, AB Canada; 3 Nashville Biosciences Nashville, TN United States

**Keywords:** external control arm, synthetic control arm, single-arm trial, real-world evidence, regulatory approval, data, clinical, decision-making, efficacy, rare conditions, trial

## Abstract

The preferred evidence of a large randomized controlled trial is difficult to adopt in scenarios, such as rare conditions or clinical subgroups with high unmet needs, and evidence from external sources, including real-world data, is being increasingly considered by decision makers. Real-world data originate from many sources, and identifying suitable real-world data that can be used to contextualize a single-arm trial, as an external control arm, has several challenges. In this viewpoint article, we provide an overview of the technical challenges raised by regulatory and health reimbursement agencies when evaluating comparative efficacy, such as identification, outcome, and time selection challenges. By breaking down these challenges, we provide practical solutions for researchers to consider through the approaches of detailed planning, collection, and record linkage to analyze external data for comparative efficacy.

## Introduction

Historically, real-world evidence from observational studies has had limited use for demonstrating therapeutic effectiveness for regulatory and reimbursement purposes. However, several recent developments, including the 21st Century Cure Act, increased accessibility to large-scale routinely collected health data, improved standardization of collection, and increased high-profile regulatory applications, and have resulted in an increased demand for these data and increased availability of these data [1,2]. This has changed the data landscape, with growing recognition of the value of using real-world evidence that is applicable for regulatory and reimbursement purposes. Frameworks from regulatory bodies, such as the United States Food and Drug Administration (FDA) and European Medicines Agency (EMA), and reimbursement agencies, such as the National Institute for Health and Care Excellence (NICE), specifically call out the use of external data sources in conjunction with single-arm evidence, particularly in rare diseases or clinical subgroups where there is a high unmet medical need and traditional randomized controlled trials (RCTs) may not be feasible [3-7].

Real-world evidence is derived from rigorous analyses of real-world data. Real-world data are data related to patient health status or delivery of health care outside of RCTs where sources commonly originate from electronic health records (EHRs), medical claims data, and product and disease registries [8]. In addition to what may be considered common forms, real-world data from outside of traditional medical charting, including data from mobile phones, wearables, and patient-reported outcomes, have provided an abundance of data, allowing comprehensive capture of the natural course of a disease from both the physician and patient perspectives [9-11]. Although outside of the scope of this viewpoint, data from historical clinical trials have been found to be influential in comparative efficacy analyses and have been found to have a role in supplementing an external control study through hybrid study designs [12,13].

**Figure 1:**
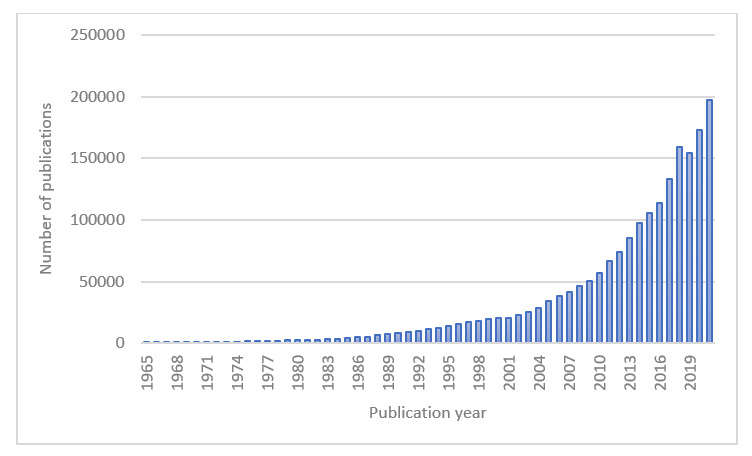
Number of real-world data and real-world evidence publications over time from 1965 to 2021. The following search terms have been considered in PubMed: “real-world” or “observational” or “nonrandomized” or “standard of care” or “external control” or “single-arm” or “historical-control” or “retrospective” or “noninterventional” or “case series” or “natural history” or “electronic health record” or “electronic medical record” or “claims”.

Given the adoption and use of EHRs and other data content sources in general practice, real-world data and real-world evidence are being increasingly reported in publications in recent years (Figure 1) and are being increasingly used in health care decisions [14,15].

As the majority of criticism from regulatory and reimbursement agencies on applications using real-world data has so far focused predominantly on data features (type, quality, and frequency) and confounding and selection bias limitations, appropriate curation and evaluation of these sources are critical components of any exercise using external evidence [16]. In this paper, we provide an overview of the challenges in identifying data suitable for external control arms, including common pseudonyms, such as synthetic control arms and historical controls, when evaluating comparative efficacy, and provide solutions for researchers to consider.

## Technical Challenges

### Challenges in Data Source Identification for Rare Conditions

RCTs have long been considered the gold standard approach to evaluate the comparative effectiveness of a drug or biological product due to the standardized methods to reduce bias, including randomization and blinding, as well as balancing known and unknown confounders. These gold standard trials, however, may not reflect the real-world setting where the patient is treated [17]. This is particularly notable for rare diseases, where the standard of care may be highly variable and disease definitions may change rapidly, than for nonrare indications. In the United States, a rare disease is defined as a condition that affects fewer than 200,000 individuals, with most diseases presenting in children or being oncology indications. Advancements in precision medicine have changed the rare disease paradigm, with many common diseases now being considered rare diseases by further splitting into subdiseases or considering stratification by mutated genes in common cancers [18]. Research focused on rare diseases is further challenged due to limited patient recruitment and siloed efforts focused on a singular therapeutic area [19,20]. Although common diseases and cancer indications are not safeguarded from similar complexities, they can often be more acute and rate limiting in the rare disease space. Regardless, both areas have their own unique technical challenges for researchers to broach throughout the life cycle of comparative efficacy analyses.

Computational models from machine learning and artificial intelligence have experienced success in early disease diagnosis and trial recruitment across several indications [21,22]. Challenges in trial recruitment have led researchers to generate computational models for patient identification, but they have faced difficulties due to lengthy diagnosis procedures, multiple physicians, and lack of gold standard–confirmed diagnoses to train models [23]. In scenarios where there is interest in rare diseases with a long disease course, these challenges can often be exacerbated due to the need for sufficient long-term follow-up data. Efforts to increase the use of data sharing in rare diseases, such as the use of FAIR (findable, accessible, interoperable, and reusable) Guiding Principles for implantation networks (eg, Global Open FAIR Implementation Network), aim to produce a conjoined effort to create data sources fit for translational research [24]. In many instances, patient identification within EHRs begins with the use of existing standard terminologies, such as the International Classification of Diseases (ICD) and the Systematized Nomenclature of Medicine – Clinical Terms (SNOMED CT). For many common comorbidities, these coding strategies have been adopted into general administrative practice but have fallen short when classifying rare diseases. A study evaluating 6519 rare diseases considered the International Classification of Diseases, 9th Revision, Clinical Modification (ICD-9-CM), International Classification of Diseases, 10th Revision, Clinical Modification (ICD-10-CM), and SNOMED CT, and found varying levels of coverage matching to a unique rare disease at 62%, 73%, and 85%, respectively [25]. With diseases being mapped to multiple ICD codes, a single representation may not constitute an accurate diagnosis. Increased coverage of rare diseases in coding languages has been acknowledged and incorporated in the International Classification of Diseases, 11th Revision (ICD-11) released in 2022, with 10 times more rare disease classifiers than the previous version [26]. Additional challenges related to executing machine learning and artificial intelligence initiatives arise due to several ethical and legal concerns, including consent for data use, transparency of algorithms, liability, and cyber security [27,28]. Given the adaptive nature, the FDA has released guidance and a proposed regulatory framework, mainly in relation to medical devices and decision support software, to enable development of these technologies while maintaining safety and oversight for transparency and performance [29,30]. Equivalent guidance has yet to be established for similar initiatives involving comparative effectiveness studies using real-world data. Similarly, advanced procedures for the diagnosis of rare diseases, such as whole genome sequencing (WGS) and advanced imaging, may not be sufficiently captured within EHRs. Initiatives surrounding WGS in particular are being developed to improve the storage, knowledge, and presentation of genomic information within EHR systems to increase use [31]. Despite the increase in coverage, the implementation of these coding strategies, conversion of previous ICD codes within EHR systems, and incorporation of advanced technologies will add an additional level of complexity for EHR-based studies.

Outside of traditional rare diseases, adoption of real-world data for comparative efficacy remains underutilized in certain categories, such as the incorporation of medications or surgical procedures in clinical trial conduct [32]. Similarly, the ability to replicate clinical trials based on real-world data alone continues to be an area to improve, as many replication failures can be attributed to analyzing endpoints not typically found in real-world data, requiring data that are unlikely to appear in a structured form, lack of complete medical history, or difficulties in closely emulating a placebo [33,34]. From herein, we focus on the following common challenges: outcome and covariate challenges, follow-up, time selection, and geography.

### Outcome and Covariate Challenges

In a traditional clinical trial, there are frameworks present for outcome ascertainment and frequency through defined protocols for the monitoring and timing of key observations to evaluate clinical effectiveness. Given the structured nature of data from RCTs, analyses using these are seldom hampered by high levels of missingness or stochastic outcome measurements. In the context of real-world data and external control arms, a common challenge encountered is addressing the mindset of researchers to be open to the value of real-world data and the ability to develop suitable outcome proxies due to insufficient data availability.

EHR systems were not designed for use in the same structured trial environment for clinical effectiveness studies; therefore, additional steps are required to refine data sets for use. EHR data include routinely collected measurements from general practice, which may not exactly match defined clinical endpoints of interest or disease definitions as encountered in clinical trials. Accordingly, where analyses attempt to align these data for comparative efficacy, issues may arise. In these cases, plausible proxies need to be made. For example, a trial of interest in a hepatology setting might include an outcome for “ascites requiring treatment;” however, in EHR data sets, “ascites” might be captured, but the condition with the additional criteria of requiring treatment would not be preassembled and indexed. Indeed, it would be possible to generate the treatments for ascites and tie these back to an individual patient, but this may be a manual process. As such, estimating the total number of eligible patients with the outcome of interest may be subject to substantial manual data curation. Specifically, in the context of using EHRs for comparisons to data obtained from a prospective clinical trial, there may be an absence of overlapping outcomes. Clinical trials (randomized or single-arm) have been found to often capture data on outcomes not recorded routinely in clinical practice [34].

Similarly, the translation of many gold standard trial endpoints to real-world data initiatives may not always be a straightforward endeavor. These efforts require a series of methodological considerations during the planning, abstraction, and analysis phases to ensure regulatory-grade fit-for-purpose data [35]. Even where data may be recorded and decisions made consistent with clinical trials, there may be differences with respect to the timing and process of testing, which may influence the availability of testing. In clinical trials of solid tumors, the Response Evaluation Criteria in Solid Tumors (RECIST) are often used to define endpoints of progression-free survival (PFS) or objective response. However, the ability to operationalize the RECIST for retrospective analysis can be hindered due to lack of consistency in imaging reports in community practices [36]. The RECIST are partially qualitative measurements, and physicians or patients in clinical practice are not blinded to the treatment, providing another difference from a clinical trial environment. Where clinical trials may make assessments for progression on a scheduled basis, real-world settings often only do so when there is an indication of progression, generally resulting in a misalignment of timing when attempts are made to draw comparisons. The real-world equivalents of many clinical trial endpoints, such as PFS and objective response rate, may not be generated using the same level of standardization or may result in variations in assessment criteria between diagnosing physicians in comparison to when performed within a clinical trial setting. For indications with good survival, such as early stage breast cancer, surrogate endpoints have been influential in allowing for reduced development time, smaller patient populations, and shorter follow-up time [37]. Surrogate endpoints, such as pathologic complete response, would require the same level of attention as long-term outcomes to adequately define a real-world equivalent. As many surrogate endpoints for topics, such as oncology, remain controversial due to questions over their direct correlation to assess patient survival, their applications from real-world data are subject to similar criticism [38]. Real-world data–based surrogate endpoints require thorough validation and can often involve timely procedures to abstract adequate data for ascertainment [39].

Other endpoints of interest, such as overall survival, may be clearly definable within a given data set, but are subject to limitations such as use restriction, delayed data availability, and missingness [40]. For example, in acute lymphoblastic leukemia, the concept of “fit-for-use” EHR data was evaluated by assessing the data suitability prior to examination of any survival-based outcomes [41]. Using a defined data quality assessment framework, data variables, such as diagnoses and demographics, were extracted and defined from EHRs over 70% of the time, but the approach fell short when categorizing laboratory values and death data, resulting in a high degree of missingness for these variables [41]. As such, when considering data missingness, it is important not to simply restrict the evaluation to availability versus nonavailability of data, but also consider the frequency of assessments and overall data suitability. While much of the current literature has focused on oncological examples and survival-based outcomes, similar considerations can be granted to other outcomes, such as adverse events, and assessment of the balance between efficacy and clinical benefit.

### Follow-up

Real-world studies provide an opportunity to offer additional insights into the patient journey by providing a reflection of traditional care. In an RCT setting, long-term follow-up can be constrained by financial hurdles and trial logistics. To mitigate this, randomized trials have been extended by incorporating long-term follow-ups augmented using routinely collected health care data to investigate long-term outcomes [42]. In these contexts, there may be somewhat broader observation windows than in traditional RCTs, and there are still predefined study visits, follow-up criteria, and study investigators ensuring consistency of follow-up. In contrast, patients in general practice demonstrate higher variability with respect to their follow-up times and their frequency of follow-up visits. Patients’ frequency of visits may be heavily correlated with the severity of their condition or diagnosis, resulting in potential biases for follow-up times. Specifically considering this in the context of time-to-event outcomes, imbalances in follow-up frequency can result in biased estimations of relative treatment effects [43]. Empirically, this has been demonstrated with observational data, and showing the level of follow-up completeness has been influential in the accuracy of survival estimates [44]. In this example, simple reporting standardization of study follow-up, including in general practice, was found to be influential in outcome assessments, with a lack of systematic follow-up resulting in an underestimation of mortality [44]. Similarly, the frequency of assessment scans has been shown to be associated with higher median PFS for both treated and untreated populations [45]. As such, where assessments of PFS are made using real-world data with low or high frequency of assessments relative to the comparator data, the potential for bias may be high. In addition to visit frequency, the United States has additional hurdles contributing to the lack of sufficient follow-up data due to clinicians experiencing a higher administrative burden when using EHR systems in comparison to other countries, discontinuity resulting from out-of-system care, and a direct relationship with enrollment in health care insurance [46-48]. The value of incorporating real-world data into a framework to be considered as evidence for future extension studies has been recognized and endorsed by agencies such as the International Society for Pharmacoepidemiology [49].

### Time Selection Challenges

Unlike in randomized controlled comparative efficacy studies, the definition of start date for measuring patient outcomes can be challenging in routinely collected health data. The concept of immortal time bias originated in the 1970s and is termed to account for the period of time within an observational or follow-up period of a cohort where the study outcome of interest cannot occur [50]. When unaccounted for, EHR studies can be prone to immortal time bias, and this phenomenon has been demonstrated in observational studies of long-term conditions [51]. For example, clinical trials may often be established to evaluate a therapy within a population having an existing therapeutic history, which is often referred to as a line of therapy. There may be limits with respect to the total lines of therapy, but they are otherwise permissive with respect to treatment lines or may even use treatment lines as a stratification factor. In routinely collected health data, this presents a challenge for patients with multiple lines of therapy, who are “multiply eligible” for the trial of interest. For a patient who has received 4 lines of therapy, which line of therapy should be considered their index date? Patients could be selected according to their most recent line of therapy or oldest line of therapy, or there could be a random selection of the therapy line. Each of these may have an influence on the associated outcomes, particularly those that may have a time-varying frequency. Hernan and Robins proposed several approaches within the context of target trial emulation. It has been proposed to use a single time zero, a randomly selected time zero, or all available time zeros for a given patient [52]. Within the context of a comparative efficacy analysis using external trial data, these approaches may not be as interchangeable. Indeed, simulation-based research in an oncology context identified that the use of either random line selection or the last line of therapy was subject to substantial inflation in type I error when compared to the use of all available lines of therapy [53]. As such, careful selection of the index date should be applied as decisions may substantially alter the associated inferences available.

Related to these issues are those representing general temporal biases associated with discordant timeframes of interest. While statistical methodologies may be used to minimize observable between-group differences, other aspects of care may vary over time in ways that cannot be accounted for in such a direct manner. For example, even where diagnostic criteria for a condition do not vary over the time period of interest, access to the requisite diagnostic technology may increase over time, leading to increased disease incidence outside of any other changes to practice. These patients may differ from patients identified at different times with respect to both measurable and unmeasurable characteristics, which may contribute to temporal bias [54]. This is separate from more clear-cut temporal bias, which may occur when diagnostic criteria change for patients, standards of care vary over time, or important prognostic factors of significance are identified, and it would need to be adequately accounted for. Finally, these changes in practice over time may result in essential covariates or populations of interest being simply unavailable owing to evolution of the standard of care.

Indeed, concerns regarding temporal bias have formed the basis of negative reception in regulatory submissions using external data for the FDA, the EMA, and Health Canada [55,56]. Statistical approaches exist to identify time-varying confounding [54], though these are predominantly for exploring the existence of such confounding, rather than to minimize their impact. As such, where possible, it is important to achieve close alignment with respect to the timeline of interest, and where this is not possible, it is important to identify covariates that may influence the differences in dates.

### Geographical Context

Conditional on the circumstances associated with the application of an external control, geographic representation may be an important characteristic to consider. Regulatory and reimbursement bodies have often identified geographic discordance as part of the rationale behind negative endorsement of candidate products, particularly where these geographic differences are likely to translate to differences in prognostic factors, confounding, effect modifiers, and standards of care [57]. Management of these issues can be minimized through ensuring specificity of the geographic source of the data of interest to ensure consistency with the target trial of interest. This may not be possible in all instances. Real-world data may be abundant in high-income countries (eg, countries in North America and Europe) but can be significantly lacking in many low- and middle-income countries due to lack of EHRs, agreement between stakeholders, and regulation to support the generation and use of secondary data [58]. The commercialization of data from many high-income countries has made identification more attainable, but given that there is no organization dedicated to the tracking of sources in low- and middle-income countries, barriers remain for researchers to identify these sources with ease [59]. Further compounding these issues is the substantial variations with respect to the availability of data in certain geographies owing to the varying legal frameworks associated with the use of patient data and differences in language. Efforts to address the ability to generalize study inferences outside of the intended study population of a geography to another is an emerging topic of interest known as transportability, and it has shown success under special circumstances [60,61]. Despite advancement, geography remains a concern for researchers to contend with when wanting to have sufficient population coverage across high- and low-income countries.

## Solutions: How Can Challenges Be Minimized?

### Solution 1: Transparent Prespecified Description of Data Element Definitions and a Detailed Data Analysis Plan

The abundance of real-world data certainly presents a vast set of challenges, including “data dredging” where the hoped-for result may stem from ad hoc data mining or from selection of one of the many data sources with limited characteristics for adjustment [62-65]. With the large quantities of available real-world data across the United States and Europe, data sets can conceivably be stratified and matched in ways that could provide favorable results in an opaque manner. To mitigate this, solutions have been presented by regulatory bodies and academic groups for improving transparency for comparative efficacy exercises using real-world data. FDA guidance specifically calls out that study design elements, including data source definitions of all data elements and analyses, should be prespecified prior to analysis, and encourages groups submitting to partake in preanalysis discussions early in the drug development program about whether conducting an externally controlled trial instead of an RCT is reasonable [7]. Academic groups, such as the International Society for Pharmacoeconomics and Outcomes Research (ISPOR) task force, also have guidance on good practices for real-world data studies of treatment effectiveness [66]. The provided advice is to have a transparent and systematic review process of combing through the data source availability and matching to trial characteristics.

The intended steps of using real-world data to evaluate clinical efficacy should be discussed or described in a protocol and statistical analysis plan (SAP) with the associated regulatory or reimbursement body ahead of initiating the externally controlled trial, in an effort to not “cherry pick” the results. Here, sponsors should include a justification for selecting or excluding relevant data sources and should demonstrate that the choice of the final data source for the control arm best answers the research question of interest [7]. Further, it would be imperative to describe how the relevant EHR data were extracted and imported into the sponsor’s electronic system, and how the data obtained from EHRs are consistent with the data collection specified in the clinical trial protocol.

FDA guidance specifies that the protocol and SAP that will be submitted following initial discussions should describe the data provenance curation and transformation procedures in the final study-specific analytic data set and describe how processes could affect data integrity (consistency of data and completeness) and overall study validity [3]. Given the origin of real-world data, SAPs should also specify all proposed sensitivity analyses or quantitative bias analyses as suggested by FDA guidance documentation to address the influence of outcome misclassification and unmeasured confounders in order to ensure appropriate conclusions are drawn [3,7]. Taken together, prespecifying data source collection methods, data element availability, and data integrity to regulatory authorities before conducting any analyses can provide transparency regarding the upcoming efficacy analyses and provide a solution to data mining.

One approach to doing this is to define the patient population for the external control arm, specify the outcome of interest, identify prognostic factors associated with the outcome of interest, and specify the control therapy. During the data source identification phase of the study, it may be common to undertake a scoping exercise to understand the breadth of data available and decipher top-level patient counts. From these selection criteria, data sets that do not have available data for elements of interest can be excluded. The derived top-level counts will reduce as further matching with trial selection criteria is applied. It is an iterative process to assess the best data source that will have sufficient patient counts and covariates to confirm a match, by assessing the strengths and limitations of each of these data sources simultaneously. Since the identification of a suitable real-world data database is an iterative process when proposing the external control arm to regulators at the time of filing for regulatory discussions, a prespecified description of the database is suggested to inform regulators, but a major limitation is that some data element criteria might be unknown until further exploration [7]. Among included data sets, assessments may be made for sample size estimates of the closest resemblance to the effective population and data missingness. The data sets with the highest effective sample size and better coverage of data elements are considered for a feasibility assessment with a deeper look at the data. Through tokenization or other methodologies, multiple data sources could also be used to get more complete patient health care data, follow-up information, and geographic coverage.

### Solution 2: Data Collection Leveraging Real-World Data

Situations where existing data are not available or suitable for an indication, a necessary exposure, an outcome, or a key covariate to measure confounding from sources, can pose a major limitation to conducting comparative efficacy studies to support regulatory decisions. As other researchers have stated, a common external control arm critique is about the mitigation of confounding [16]. Unfortunately, there are limits to ascertain real-world data proxies for variables in historically collected databases. This could also be problematic when a group may wish to minimize temporal bias due to changes in the standard of care when using older data, by restricting the dates of eligible patients. This in turn could result in low sample sizes of well-matched control patients.

A possible solution for these issues is to conduct de novo retrospective data collection where there would be manual review of patient charts, in conjunction with pre-existing data. Here, clinicians and key experts for the indication create a customized data collection form (also known as an electronic case record form) that can be standardized across multiple sites and gather useful details. In these scenarios, assessments can be obtained and linked directly to events of interest, such as a new medication or an annual physical examination. As EHR databases can provide access to longitudinal data, further evaluation of measures both before and after the event window can be performed. Drawing upon these aspects further emphasizes the value of real-world data to contribute to both predictive analytics and assessment of long-term outcomes in comparative effectiveness analyses.

De novo data capture will not fix challenges in relation to the frequency of visits or collection of variables known to exist exclusively within trial settings, which is a limitation of this solution. However, qualitative assessments and clinical narratives can contain valuable insights absent from the structured data fields of EHRs. Improvements in advanced analytics and natural language processing have provided increased automation to abstract valuable clinical information from unstructured fields, which is traditionally time consuming. A direct comparison between machine learning review and chart-based review in the diagnosis of rheumatoid arthritis demonstrated the ability of the algorithm to identify patients with a strong overlap in disease classification criteria and baseline disease characteristics [67]. On the contrary, missing data and poor data quality can introduce bias in automated methods, and variability in models can limit comparisons [68,69]. Commercial data providers have realized the potential of custom abstraction projects incorporating both manual and automated abstraction to increase the availability of data outside of structured fields, as well as algorithms to calculate real-world endpoints in product offerings.

### Solution 3: Record Linkage/Tokenization

The ability to characterize the entire patient journey is critical to perform clinical effectiveness analyses using real-world data, though a complete picture of a patient’s medical health is often not available within a single data source. Patients who move in and out of health care networks could have their data omitted from analyses of single data sets. If this movement is related to the patient’s standard of care or access to treatment, the process can result in unintended biases in an analysis that is unable to account for these patient movements and missing data. A solution to the fragmentation of the patient journey and perspective could be to engage with data providers who tokenize their data set. In tokenized data sets, patient “tokens” or unique identifiers are assigned to link a patient within a given data source to assist identification in other separate real-world data sources, avoiding duplication while protecting patient privacy at the same time [70]. Tokenization and record linkage can aid in this endeavor and have been recognized by regulators through inclusion in draft guidelines to provide considerations when performing data linkage to ensure a comprehensive data set, quantification of errors, and resolution of discrepancies [3]. Similarly, initiatives have been developed to combine machine learning methodologies and tokenization to expand upon use for EHR analysis, while drawing attention to areas needing improvement in patient phenotyping or patient identification for research purposes [71].

Examples of the role of tokenization have been identified for broadening data sets to improve associations of testing with outcomes in diseases such as COVID-19 [72,73]. While these approaches may be applicable in select cases, it is important to note that not all data sources are capable of linking data due to privacy concerns regarding indirect personal data and data ownership, and additional challenges due to differences in language. These themes highlight the potential limitations that can arise when considering linkage and tokenization to aid in downstream analyses. In turn, this necessitates a flexible approach to data identification and consideration of which approaches of further data acquisition are available for a given project.

## Summary of Real-World Data Identification Challenges and Case Studies

Every scenario in identifying a suitable real-world data source as an external control arm for comparative efficacy is unique. The solution of tokenization, for example, could work in some instances, but might not be appropriate in cases where complete coverage of data is available, though the definitions in the data set are poorly defined in the external data source. Table 1 lists the challenges that frequently arise in these data landscaping exercises and summarizes some examples of solutions or case studies for further reading.

**Table 1:** Summary of real-world data identification for comparative efficacy using externally controlled trial challenges, examples, and application of solutions.

Challenges			Examples		Application of solutions
**Data source identification of rare conditions**					
	Indecision gaps due to abundance of real-world data	It is difficult to parse out important data sources, rare disease candidates, and data linkage options.		Machine learning applications can improve accuracy and quality (type and frequency) in data source selection and patient selection [23,67,71]	
**Outcome and covariate**					
	Poorly defined variables or inconsistent definitions from clinical trial to real-world data for limited comparability of real-world data	The conceptual definition of a data element does not align with the operational definition.		De novo data collection [74] Automated electronic health record (EHR) abstraction [69] Characterization of real-world variables or surrogate endpoints [75] Prespecify sensitivity analyses, including quantitative bias analyses [76], in the statistical analysis plan	
	Medical claims data might have limited use to support regulatory-grade decision-making	Claims data have limited clinical outcome data.		Combine with EHRs to expand the applicability, coverage, and depth of data [77]	
**Follow-up**					
	Difficult to capture continuity of care in a single data source	Diagnosis is spread across multiple physicians; if the patient moves and seeks care outside of the care network, follow-up data will be lost.		Tokenization/data linkage and advanced analytics with EHR data for capturing a more complete patient journey (particularly helpful for rare conditions where the sample size would be low) [23,44,71] Analytical approaches (ie, imputation) for missing data [23,44,71,78]	
**Time selection**					
	Timing of therapy	Patient has multiple lines of treatment; what should be considered the index date?		Define a proper index date or “time zero” following the target trial emulation framework [52]	
	Timing of data collection – inconsistent standard of care over time	Data may be present, but are not current enough to provide a reasonable comparison to the current standard of care.		De novo data collection [55] Tokenization/data linkage [78]	
**Geography**					
	External control arm nongeneralizable to clinical practice	Geographic representation where the main external control arm data source is from outside of the country of interest. Select two unlinked data sources with available data to obtain a sufficient sample size. However, it is unclear if patients overlap in care networks.		Tokenization/data linkage, which improves patient counts with geographic representation while accounting for duplicates [79] Transportability [60]	
**Analysis phase**					
	Data loss or insufficient sample size to detect power	In the analysis phase, during matching, the power to detect an effect is reduced.		De novo data collection [55] Tokenization/data linkage [23,44] Analytical approaches (ie, imputation) for missing data [78]	
	Avoid the appearance of the analysis as post-hoc or cherry picked	Data dredging/post-hoc analysis (eg, regulators can assume the most appealing analysis was conducted).		Transparent prespecified description of data selection, data provenance, and the statistical analysis plan [3]	

## Conclusion

Given the considerable costs of novel drug development pipelines and the increasing stratification of diseases and subtypes, real-world data will become increasingly relevant for regulatory and reimbursement discussions in the decades to come. Exclusive reliance on an RCT framework as the entire evidence generation plan does not adequately acknowledge the shortcomings of a singular research strategy, despite the advantages for comparative efficacy research. Simultaneously, the field of biostatistics has expanded to focus on data missingness to allow effectiveness analyses when a subject presents with incomplete data, while taking the necessary precautions and accounting for bias.

The versatility of the use of real-world data extends beyond its use in comparative efficacy analyses. There are additional concerns with safety, which have postmarket authorization requirement differences. Similarly, important topics outside the scope of this paper include the many challenges associated with patient privacy and reidentification risk, and the necessary consideration needed when performing these analyses. EHR-based real-world data were the focus of this paper, but additional sources have been successful in comparative efficacy analyses. Claims, like other sources of real-world data, have their own unique challenges for consideration [80], but have demonstrated success in clinical effectiveness studies and in rare disease studies [77,81,82]. Conjoined efforts, such as those between the ISPOR and the International Society for Pharmaceutical Engineering, have provided recommendations to highlight the need for transparency in planning and reporting of observational real-world evidence studies and comparative effectiveness studies [66]. Although real-world evidence based on real-world data studies is critical to the operation of providing timely insights into what works for patients and when, the identification and evaluation of real-world data sources for comparative effectiveness studies have many challenges. The solutions suggested in this paper could minimize these challenges; however, the selection and evaluation of a good real-world data source is not as straightforward as it may appear.

## Abbreviations

EHR: electronic health record
EMA: European Medicines Agency
FAIR: findable, accessible, interoperable, and reusable
FDA: Food and Drug Administration
ICD: International Classification of Diseases
ISPOR: International Society for Pharmacoeconomics and Outcomes Research
PFS: progression-free survival
RCT: randomized controlled trial
RECIST: Response Evaluation Criteria in Solid Tumors
SAP: statistical analysis plan
SNOMED CT: Systematized Nomenclature of Medicine – Clinical Terms
WGS: whole genome sequencing
